# Supplementary material for: Human Wharton’s Jelly-Derived Mesenchymal Stem Cells Minimally Improve the Growth Kinetics and Cardiomyocyte Differentiation of Aged Murine Cardiac c-kit Cells in In Vitro without Rejuvenating Effect
Source: Int J Mol Sci. 2019 Nov 6;20(22):5519. doi: 10.3390/ijms20225519 (PMC6887783; doi:10.3390/ijms20225519)
Supplement: Supplementary file 1 [file ijms-20-05519-s001.pdf]

Supplementary Information

Cardiac c-kit Cell Complete Growth Medium (CGM) was made up of two solutions: Solution 1 and Solution 2. Solution 1 comprised of DMEM/F12 containing 1% (v/v) insulin-transferrin-selenium, 1% (v/v) Penicillin-Streptomycin, 0.1% (v/v) fungizone and 0.1% (v/v) gentamicin. Solution 2 comprised of Neurobasal medium supplemented with 37 mg of L-glutamine, 2% (v/v) B27 supplement and 1% (v/v) N2 supplement. The complete growth medium was prepared by mixing the solutions in the ratio: 45% solution 1, 45% solution 2 and 10% (v/v) embryonic stem cell-qualified FBS (All the above were purchased from Gibco®, Invitrogen Life Technologies Co., CA, USA). Finally, CGM was supplemented with 20 ng/ml epidermal growth factor, 10 ng/ml basal fibroblast growth factor and 10 ng/ml leukemic inhibitory factor (All the growth factors were purchased from Peprotech, Rocky Hill, NG, USA). The media was then sterilised through a 0.22 m pore filter into a sterile container and store at 4 °C

Cardiomyocyte Differentiation Medium (CDM) was composed of -MEM (Sigma-Aldrich, St Louis, MO, USA) supplemented with 10% (v/v) FBS, 1% (v/v) Penicillin-Streptomycin, 0.1% (v/v) gentamicin, 0.1% (v/v) fungizone, 1 M dexamethasone (Sigma-Aldrich, St Louis, MO, USA), 50 g/ml ascorbic acid (Sigma-Aldrich, St Louis, MO, USA) and 1 mM -glycerophosphate (Sigma-Aldrich, St Louis, MO, USA).

Mesenchymal Stem Cell Growth Medium (MGM) was composed of DMEM/LG, supplemented with 1% (v/v) Penicillin-Streptomycin and 10% (v/v) FBS.

M-buffer was made up of Dulbecco Phosphate Buffer Saline (DPBS) and supplemented with 200 mM Ethylenediaminetetraacetic acid (EDTA) and 1% FBS.

Incubation Medium was made up of DPBS supplemented with 5 mg/mL Bovine Serum Albumin (BSA), 2 mM EDTA, 1x Penicillin-Streptomycin, 0.1% (v/v) gentamicin and 0.1% (v/v) fungizone.

CHAPS Lysis Buffer was composed of 10 mM Tris HCl (pH7.5), 1 mM magnesium chloride, 0.1 M PMSF, 5 mM -mercaptoethanol, 0.5% (w/v) CHAPS and 10% (v/v) glycerol.

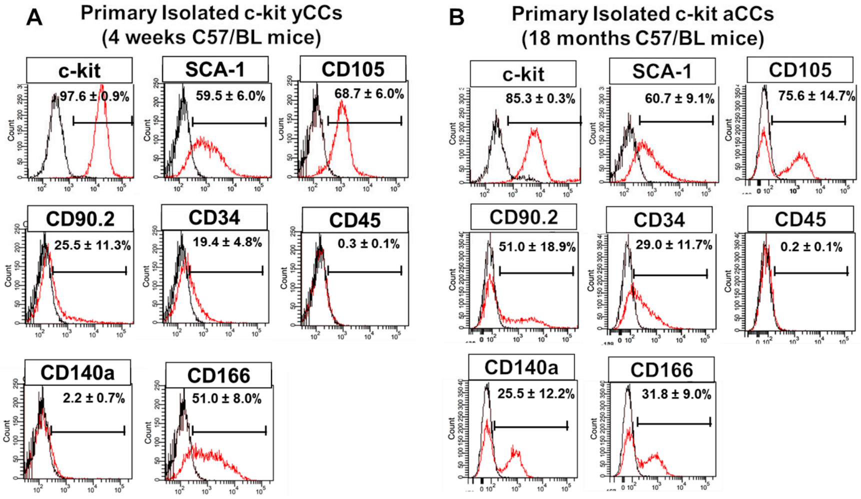

Figure S1. Multipotent CCs can be isolated from 1-month and 18-month old C57BL/6N mice. Flow cytometry analysis of c-kit-expressing CCs isolated from (A) young (1-month) and (B) aged (18-months) C57/BL6N mice. [Abbreviation: CD34, hematopoietic marker; CD45, mast cells;

CD90, thymocyte differentiation antigen; CD105, endoglin; c-kit, stem cell growth factor receptor; CD140a, PDGFR ; Sca-1, stem cell antigen; CD166, ALCAM].

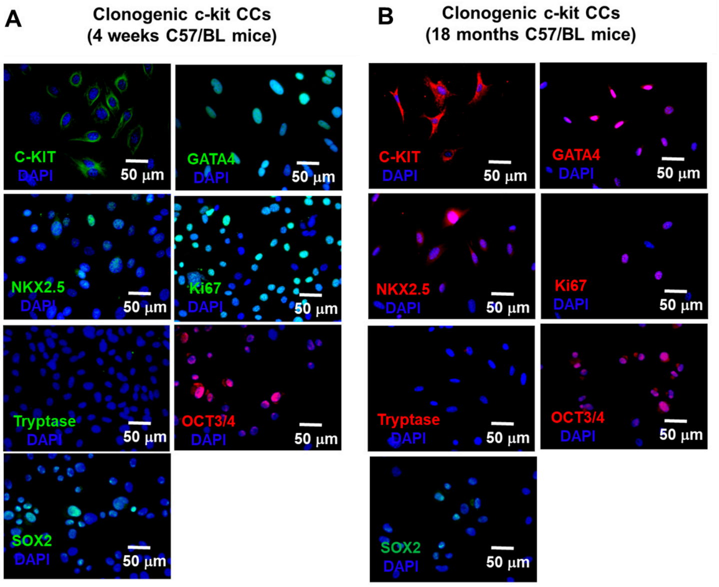

Figure S2. Immunocytochemistry staining of clonogenic CCs. Both (A) young and (B) aged CCs expressed GATA4, NKX2.5, Ki67 but negative for Tryptase. Nuclei were stained with DAPI (Blue). Scale bar= 50 µm.

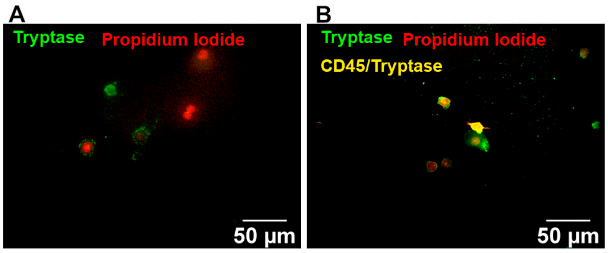

Figure S3. Immunocytochemistry staining of mast cells. (A) Mouse peritoneal mast cells were labelled with goat anti-Tryptase antibody and counterstained with AF488 tagged donkey-anti goat secondary antibody followed by (B) FITC-tagged mouse CD45, which was counter-labelled with AF546 tagged donkey-anti mouse secondary antibody. Scale bar= 50 µm.

Table S1. Mouse-Specific Primers Validation.

Gene	Mouse Sample Ct Value	Human Sample Ct Value
Gapdh	21.3	36.65 *
Myh6	28.87	Undetermined
Myh7	26.77	35.89 *
Gata4	18.42	36.98 *
Nkx2.5	24.35	35.47 *
Tnni3	26.79	Undetermined

* Ct value >35 is considered as under detection limit in this study
